# Assortative mixing in spatially-extended networks

**DOI:** 10.1038/s41598-018-32160-4

**Published:** 2018-09-14

**Authors:** Vladimir V. Makarov, Daniil V. Kirsanov, Nikita S. Frolov, Vladimir A. Maksimenko, Xuelong Li, Zhen Wang, Alexander E. Hramov, Stefano Boccaletti

**Affiliations:** 1 REC ‘Artificial Intelligence Systems and Neurotechnology’, Yurij Gagarin State Technical University of Saratov, Polytechnicheskaja str 77, 410054 Saratov, Russia; 2 Faculty of Nonlinear Processes, Saratov State University, Astrakhanskaja str. 83, 410012 Saratov, Russia; 3 Xi’an Institute of Optics and Precision Mechanics, Chinese Academy of Sciences, Xi’an, 710119 Shaanxi China; 4 School of Mechanical Engineering and Center for OPTical IMagery Analysis and Learning (OPTIMAL), Northwestern Polytechnical University, Xi’an, 710072 Shaanxi China; 5 CNR-Institute of Complex Systems, Via Madonna del Piano 10, 50019 Sesto Fiorentino, Florence, Italy; 6 Unmanned Systems Research Institute, Northwestern Polytechnical University, Xi’an, 710072 Shaanxi China

## Abstract

We focus on spatially-extended networks during their transition from short-range connectivities to a scale-free structure expressed by heavy-tailed degree-distribution. In particular, a model is introduced for the generation of such graphs, which combines spatial growth and preferential attachment. In this model the transition to heterogeneous structures is always accompanied by a change in the graph’s degree-degree correlation properties: while high assortativity levels characterize the dominance of short distance couplings, long-range connectivity structures are associated with small amounts of disassortativity. Our results allow to infer that a disassortative mixing is essential for establishing long-range links. We discuss also how our findings are consistent with recent experimental studies of 2-dimensional neuronal cultures.

## Introduction

Spatial constraints are often the main factor shaping the structure of connections in real-world networked systems^1,2^. For instance, interaction in biological systems (such as populations of animal species) is strongly dependent on the overlap of their habitats^3^. Other examples are social networks (which frequently demonstrate spatial homophily)^4^, the patterns of disease spreading (which are strongly connected with physical contacts of individuals)^5^, or the topology of urban networks (which is almost completely determined by their spatial configuration)^6–8^.

Spatial distribution effects are however less studied on the topology of biological networks, such as neuronal cultures. The ability of these latter systems to attain and maintain an optimal connection structure, often studied on *in vitro* spatial cultures^9^, is unique. Different organization layers of neuronal networks exhibit disparate topological scales in the brain^10^ and, accordingly, distinct and diverse ranges of connectivity. For instance, while the existence of long-range interactions between the neurons via synaptic connections is nowadays well-known^11^, recent studies have also highlighted that astrocyte cells^12^ modulate neural synaptic communication via glutamate diffusion and provide slow short-range neural interactions^13,14^.

Therefore, the importance of understanding the topological properties of spatially-extended connectivity structures along with the principles beneath their formation and emergence is indubitable. So far, extensive studies have been made^15,16^, in particular for revealing the properties of spatial scale-free networks^17^, for which a wealth of numerical models^18,19^ were proposed. However, several recent evidences highlighted that spatially-extended networks do not necessarily exhibit scale-free property^20,21^: spatial social networks together with inside-country airport networks^22^ display small-world structures^23^ with Poisson-like degree distribution^24^, which intimately depends on short-range connectivity^25^.

We here investigate the assortativity properties of spatially-extended networks, during their transition from short-range to long-range connectivity structures. Our study is motivated by the existing strong relation between positive degree-degree correlations and robustness of the networks^26,27^. In particular, we will first design a model for the graph evolution, based on spatial growth and preferential attachment principles. With the help of the model, we will then show that a topological transition leads to a scale-free network structure in connection with a decreased assortativity, thus revealing that emergence of disassortative mixing is essential for establishing long-range links in spatial networks. Finally, we will discuss how our results are consistent with a series of recent experimental evidence in 2D neuronal cultures^9,28^.

## Results

A variety of models for the construction of spatially-extended networks has been proposed in the past years. Most of them follow the fact that in real networks (such as urban systems or fungal tissues) the nodes are not located randomly, and the probability of appearance of new nodes is higher in the vicinity of existing ones^29^. This evidence is accounted for by the modified correlated percolation model^30^, which assumes that the probability of appearance of a new node, *j*, depends on the occupancy of the neighborhood. At the same time, other models were proposed for the generation of spatial scale-free networks, using preferential attachment^18,19^. We here combine these two principles to obtain both spatial and structural inhomogeneity, i.e. to ensure that even nodes located at the same distances can have different probability of connection to each other, depending on their degrees.

In particular, we consider a growth process wherein new nodes are sequentially added to an existing graph, and the probability for a newly added node *j* to form a connection with an already existing node *i* is

1 $$\begin{array}{l}{p}_{ij}={e}^{-\lambda {R}_{ij}\cdot {d}_{i}^{-\beta }},\end{array}$$

where *λ* is a density gradient^30^ which defines the decrease of *p*_*ij*_ with the Euclidian distance *R*_*ij*_ between nodes *i* and *j*, and *β* is a degree factor allowing to reduce the impact of the density gradient when the degree, *d*_*i*_, of the existing node *i* is sufficiently large.

**Figure 1:**
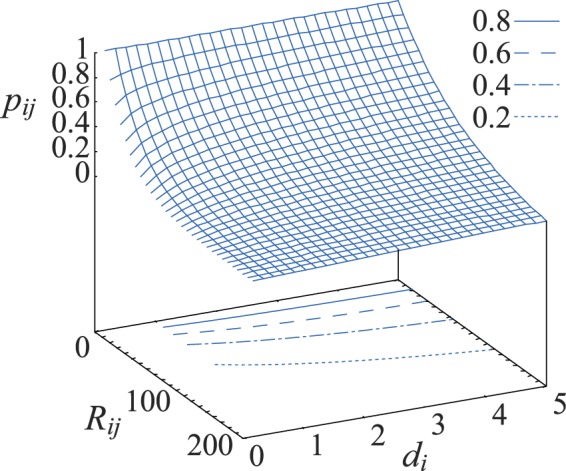
The probability of edge emergence, *p*_*ij*_, as a function of the Euclidean distance between nodes *i* and *j* (*R*_*ij*_) and of the degree of node *i* (*d*_*i*_). Several iso-probability lines are shown by contour lines on the lower surface (see legend). The parameters are here set to *λ* = 0.02 and *β* = 0.3.

Figure 1 shows how the probability of forming an edge depends on the distance between nodes, *R*_*ij*_, and on the degree, *d*_*i*_, of the existing node *i*, for moderate values of *λ* and *β*. When *d*_*i*_ is small enough, increasing *R*_*ij*_ leads to a sharp decrease of *p*_*ij*_. At the same time, growth of *d*_*i*_ extends the area of parameter space where *p*_*ij*_ is rather large, i.e. the effective connectivity range, allowing one to implement a preferential attachment rule: even those high-degree nodes which are far away in space have a relatively high chance to form connections with newly added vertices.

The algorithm for the generation of graphs is as follows. First, one assigns the number of nodes, *N*, and defines a square coordinate plane (*X*, *Y*) of size (10^4^ × 10^4^). Then, the procedure is implemented according to the following steps:

(i) One randomly assigns the coordinates of an initial node in the range [2500: 7500, 2500: 7500]. This intervals is chosen for the purpose of avoiding the effects of spatial constraints, which may constitute a bias if the initial node is placed too close to the boundaries of the coordinate plane.
(ii) The *N* × *N* adjacency matrix *W* is constructed, and initially filled with zeros.
(iii) A new node *j* is added with randomly assigned (*X*, *Y*) coordinates on the coordinate plane. Its distances *R*_*ij*_ are calculated with respect to all existing *i* nodes.
(iv) If *R*_*ij*_ is less or equal to 1 for at least one pair of nodes (*i*, *j*), then the coordinates of node *j* are reassigned, until the condition ${R}_{ij} > \mathrm{1,}\forall i$ is met.
(v) The probability *p*_*ij*_ [of connecting node *j* with any other node *i* from the set of the already existing ones] is calculated according to Eq. (1).
(vi) Random numbers with a uniform probability distribution in [0, 1] are generated, and for each pair (*i* ≠ *j*) *W*_*ij*_ = *W*_*ji*_ is set to 1 whenever a connection is formed between node *i* and *j* [i.e. whenever the generated random number is smaller than *p*_*ij*_].
(vii) After completion of step (vi), if the added node had not formed any connection with the pristine sub-graph, then one reassigns the coordinates of the node and returns to step (iv). Otherwise, one goes back to step (iii) and continues the growth process of the network until the number of nodes becomes equal to the desired value, *N*.

**Figure 2:**
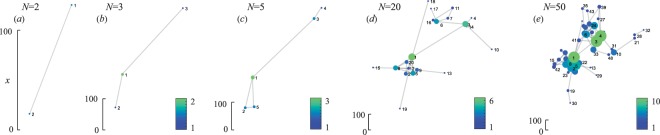
Schematic illustration of the network generation algorithm. Visualizations of the network are given at different stages of the growth (i.e. different number of attached nodes): (**a**) *n* = 2, (**b**) *n* = 3, (**c**) *n* = 5, (**d**) *n* = 20, (**e**) *n* = 50. For comparison, the spatial scale is given on each figure. The color of the node (together with its size) denotes its degree (see legends). Once again, the control parameters are *λ* = 0.02 and *β* = 0.3.

The algorithm is schematically illustrated (for *N* = 50) in Fig. 2, with the same values of control parameters as those used for Fig. 1. In panel (a), only one new node is added to the initial node (*N* = 2). The cases *N* = 3 and *N* = 5 are reported in panels (b) and (c) respectively, and correspond to the formation of a main cluster. In the last stages of the network’s growth [panels (d) and (e), where *N* = 20 and *N* = 50] the mentioned cluster is seen distinctly. Moving from each one to the next panel, the scale of the coordinate plane shrinks whereas the average node degree increases. Consequently, emergence of spatial inhomogeneity in the node density together with formation of spatial clusters are the two main observed effects, resulting eventually in an inhomogeneous degree distribution over the entire graph, and in the appearance of meso-scale structures.

To gather further insight into the topology of the obtained networks, one can rely on the estimation of a wealth of structural measures: the average degree, the clustering coefficient, the graph’s efficiency, the assortativity properties, and the algebraic connectivity. The average degree is just the sum of the degrees, *d*_*i*_, of all nodes divided by the number of nodes, *N*:

2 $$\begin{array}{l}{d}_{a}=\frac{1}{N}\sum _{i\mathrm{=1}}^{N}{d}_{i}.\end{array}$$

We use the global clustering coefficient, which present itself the average of local clustering coefficient over the network, and proposed in ref.^31^. The network efficiency is defined by:

3 $$\begin{array}{l}E=\frac{1}{2N(N-\mathrm{1)}}\sum _{i\ne j}{v}_{ij},\end{array}$$

where *v*_*ij*_ is the length (in steps) of the shortest path between the nodes *i* and *j* of the graph.

We here concentrate on degree-degree correlation, the graph’s homophilic property which quantifies the tendency of nodes to link with vertices with similar (or dissimilar) degree. For this purpose, we quantify the network’s assortativity by means of the Pearson correlation coefficient of the degree between pairs of linked nodes^32^:

4 $$\begin{array}{l}r=\frac{{M}^{-1}\sum _{k}^{M}{d}_{k}{b}_{k}-{[{M}^{-1}\sum _{k}^{M}\frac{1}{2}({d}_{k}+{b}_{k})]}^{2}}{{{M}^{-1}\sum _{k}^{M}\frac{1}{2}({d}_{k}^{2}+{b}_{k}^{2})]}^{2}-{[{M}^{-1}\sum _{k}^{M}\frac{1}{2}({d}_{k}+{b}_{k})]}^{2}},\end{array}$$

where *M* is the total number of edges, and *d*_*k*_ and *b*_*k*_ are the degrees of the nodes at the two ends of the *k*_*th*_ link (*k* = 1, …, *M*). A positive value of *r* reflects the tendency of nodes to form links with the other nodes of the graph featuring the same (or a similar) degree; a negative value of *r* indicates instead the propensity of low-degree nodes to connect to structural hubs. We furthermore consider the average degree of the neighborhood of each node *i*, defined by

5 $$\begin{array}{l}{u}_{i}=\frac{1}{{d}_{i}}\sum _{j\mathrm{=1}}^{N}{W}_{ij}{d}_{j},\end{array}$$

where *W*_*ij*_ = 1 are the elements of the adjacency matrix accounting for the existence of a link between nodes *i* and *j*, and *d*_*j*_ is the degree of node *j*. Finally, we also calculate the algebraic connectivity, which is the 2-nd smallest eigenvalue of the Laplacian matrix^33^, and has a prominent role in many relevant dynamical processes on networks, such as synchronization and diffusion.

**Figure 3:**
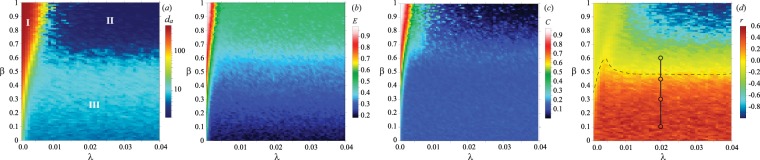
2D-plots reporting the values of several network’s topological measures in the parameter space (*λ*, *β*): (**a**) the average degree over the network (given in log scale), (**b**) the graph’s efficiency *E*, (**c**) the clustering coefficient *C*, (**d**) the assortativity coefficient *r*. See the main text for all the definitions. For each panel, the color code is reflected in the corresponding legend. The dashed contour line in panel (**d**) connects all points where *r* = 0. Other labels appearing in panels (**a**,**d**) are defined and discussed in the main text. All networks have *N* = 500 nodes.

With these stipulations, we first set *N* = 500 and investigate the global characteristics of the obtained networks while varying the model parameters *λ* and *β*. Figure 3 shows the 2D plots of the nodes’ average degree (panel a, in log scale), the network efficiency as given by Eq. (4) (panel b), the clustering (panel c) and assortativity (panel d) coefficients.

Three main parameter regions can be isolated in panel a, which are marked with “I”, “II” and “III” respectively, and which correspond to completely different emerging topologies. In the region marked by I the average degree is higher than 100, and the resulting graph is a globally or nearly-globally coupled network. This regime occurs at very small values of *λ*, where spatial embedding is almost irrelevant in that the emergence of links between nodes is practically uncorrelated to the spatial coordinates. An increase in *β* widens such a region, due to the fact that the connectivity range of higher degree nodes is more and more expanded. In region I, the clustering coefficient and the efficiency are evidently close to 1, whereas *r* is almost vanishing. Notably, the increase of the density gradient after *λ* ≈ 0.015 does not affect the structural properties of the resulting network, leading just to a denser arrangement of the nodes.

As opposed to almost global coupling structures, the second region (II) is characterized by nearly one-to-all configurations, since higher values of *β* provoke an explosive growth of the connectivity range of the first node. The clustering coefficient here is close to zero, and most of the shortest paths are realized by two steps through the central node (and therefore *E* ≈ 0.5). Notice that *r* takes large negative values in this region.

Regions I and II appear, however, to be limit cases. The most interesting (and realistic) regime is that occurring in the third region (III), and exhibiting the formation of inhomogeneous networks, as the one reported in Fig. 2. Here, the resulting graphs exhibit small, but not negligible values, of the clustering coefficient, a fact that is typical in real-world systems^?^. Both *r* and *E* do not display uniform values over this parameter region, and clearly depend on *β*. The maximum level of assortativity (*r* ≈ 0.6) is realized at small values of the degree factor (*β* < 0.3), and *r* progressively decreases while approaching region II. On the contrary, the network efficiency *E* grows with the increase of *β*, due to the emergence of long-range connections in the network, which also results in an increase of the average degree.

**Figure 4:**
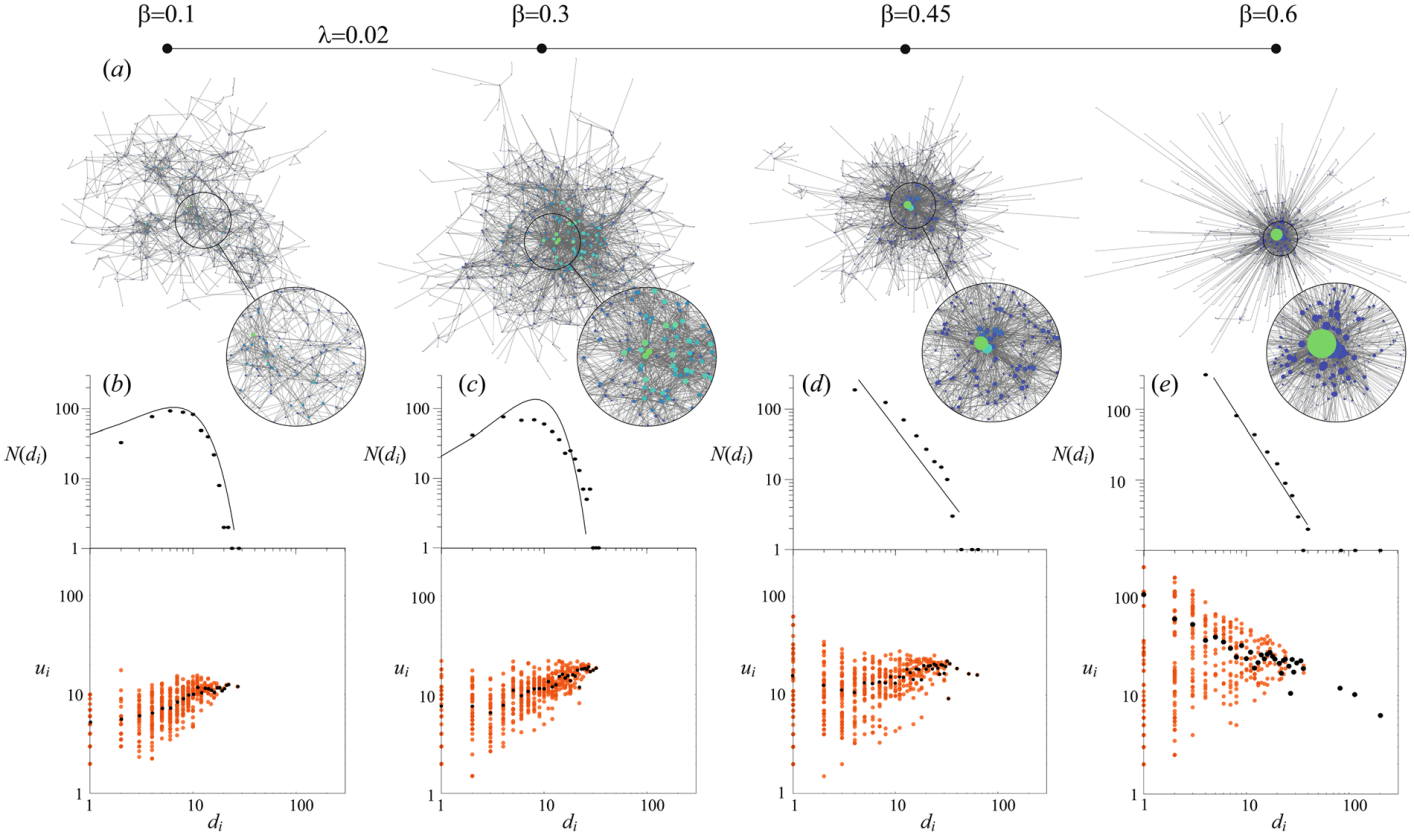
The topologies of the generated network when increasing the degree factor *β* in region III. (**a**) Spatial visualizations of the obtained network structures. The size and color of a node is proportional to its degree. The insets zoom on specific graph areas. *N* = 500. (**b**–**e**) Degree distributions (*N*(*d*_*i*_), upper plots) and correlation between *d*_*i*_ and *u*_*i*_ (lower plots) at different *β* values: (**b**) *β* = 0.1, (**c**) *β* = 0.3, (**d**) *β* = 0.45, (**e**) *β* = 0.6. The black dots on the correlation plots mark the average values of *u*_*i*_ for each *d*_*i*_ value. The solid lines in the degree distributions are marking the fit with specific distribution functions: (**b**,**c**) Poisson distribution, $N({d}_{i})=\frac{{\xi }^{{d}_{i}}}{{d}_{i}!}{e}^{-\xi }$, with $\xi =7.016$ (**b**) and $\xi =8.672$ (**c**–**e**) power law, $N({d}_{i})={d}_{i}^{-\gamma }$, with $\gamma =1.852833809$ (**d**) and $\gamma =2.296646767$ (**e**). The goodness of fit has been justified by means of one way chi square test with the following *p*-values: (**a**) *p* > 0.99, (**b**) *p* = 0.79, (**c**) *p* > 0.99, (**d**) *p* > 0.999.

In order to better understand the generation process of the network in region III, we fix *λ* = 0.02, and focus on four distinct values of *β* (marked in panel d of Fig. 3 by connected circles). Figure 4 reports the visualizations (panel a) and some characteristics (panels b-e) of the generated networks. At low values of *β* [panel a of Fig. 4] the connectivity range of the nodes is almost independent on their degree, and this gives rise to rather homogeneous spatial structures of the network with an almost constant distance between connected nodes. The lower panels of Fig. 4 reports the degree distribution of the obtained networks, and the correlation between the degree of a node (*d*_*i*_) and the average degree of its neighbours (*u*_*i*_). We justify the fitting of observed degree distributions to a particular law using one way chi square test. We find that structures obtained with a low value of *β* (such as *β* = 0.1 in Fig. 4b) brandish a Poisson degree distribution (*p* > 0.99), indicating that the obtained graph only slightly deviates from a purely random network. At the same time, however, the network exhibits assortative correlations (see the positive trend in the lower plot of panel b) caused by spatial constraints on the emergence of new connections: all nodes in some area have very similar degree.

Increasing *β* (*β* = 0.3, Fig. 4c) has two main effects. First, the network becomes more centralized, and the density increases around the initial node. Second, the degree inhomogeneity becomes more and more pronounced when moving from the graph’s center to the periphery, a fact which casts the corresponding degree distribution in a way that slightly deviates from a Poisson-like function. The latter is also shown by chi square test indicating less pronounced fit with Poisson law (p = 0.79). Strong spatial homophily of the nodes, i.e. the tendency to have the spatial neighbours with similar degree, are still present, as well as a positive network degree-degree correlation. In this region high degree nodes are capable of maintaining longer connections, but the spatial dependence of *p*_*ij*_ is not sharp enough to provoke an explosive growth of the effective connectivity of individual nodes.

The scenario changes rather dramatically when the degree factor is further increased (*β* = 0.45, Fig. 4d). The network becomes now markedly centralized, and one observes larger and larger degrees of inhomogeneity, with a degree distribution similar to a power law, $N({d}_{i})={d}_{i}^{-\gamma }$ (*p* > 0.99). *r* almost vanishes, and the loss of assortativity is caused mostly by small degree nodes which start to connect to structural hubs [see the lower plot of Fig. 4d], whereas the nodes with moderate degree still feature an assortative behavior.

Finally, when the degree factor is large enough [as the case of *β* = 0.6 reported in Fig. 4e], preferential attachment becomes dominant over the spatial constraints: those nodes with rapidly growing degrees obtain the ability to form connections at longer and longer distance, with the initial node forming connections to almost all the other nodes. Furthermore, the dominant dependence of *p*_*ij*_ on the node’s degree provokes the emergence of spatial and structural degree inhomogeneities. The degree distribution fits now very well a power law, with values of the exponent *γ* close to those featured by real systems^34^ (*p* > 0.999). The average *u*_*i*_ (black dots in the lower panel) now exhibits a negative correlation with the node degree (*d*_*i*_), as a result of the emergence of long-range links allowing hubs to aggregate the connections with peripheral nodes. At the same time, one can see that the values of *u*_*i*_ are largely spread for lower degree nodes.

**Figure 5:**
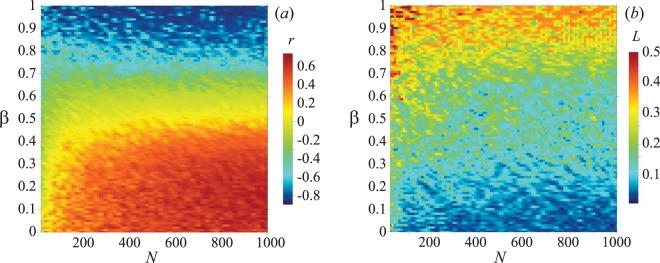
The coefficient *r* (panel a) and the algebraic connectivity *L* (panel b) in the parameter space (*N*, *β*). See main text for definitions. *λ* = 0.04.

Finally, we examine the impact of the network size. To do so, we set the value of the density gradient (*λ* = 0.04) within a stable region (i.e. where variations of the density gradient are not substantially affecting the structural properties of the graph), and change *β* and *N*. In Fig. 5a the coefficient *r* is reported in the parameter space (*N*, *β*). For small networks, the dependence of *r* on *β* is rather weak. When however *N* increases, all effects described above become pronounced and the scenario stabilizes after *N* = 400. The corresponding 2D-plot of the algebraic connectivity is shown in Fig. 5b. One can easily see that the increase of *r* is in general reflected by a decrease of the second smallest eigenvalue (*L*) of the Laplacian matrix. This, in turn, is responsible for the loss of network’s robustness, caused by the absence of long-range connections in the backbone of the graph: values of *L* close to zero indicate indeed that the network can be broken into isolated modules in a relatively easy way^35^.

## Discussion and Conclusions

In conclusion, we have revealed the existence of non trivial relationships between the spatial organization of a network and its assortativity and robustness. In particular, we have pointed out that spatial networks with strong restrictions on the connectivity range demonstrate high degrees of assortativity, and Poisson-like degree distributions. A gradual increase in the connectivity range of the higher degree nodes leads instead to the formation of long-range connections, allowing hubs to aggregate links not only with other hubs (in order to form a backbone of the graph), but also with peripheral nodes, resulting in a decrease of assortativity. At the same time, the network shapes its structure according to a scale-free topology reflected by heavy-tailed degree distribution.

**Figure 6:**
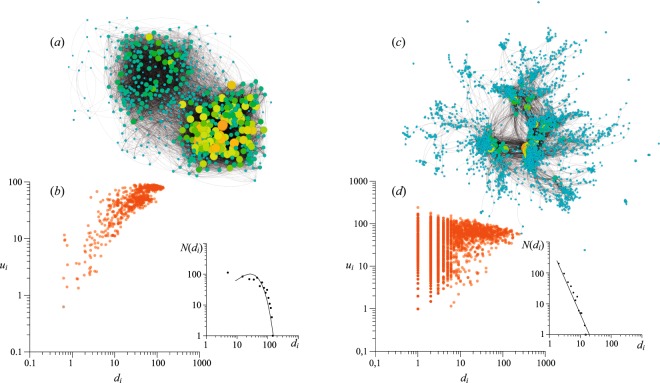
The network visualizations (**a**,**c**) and the (*d*_*i*_, *u*_*i*_) scatter plots (**b**,**d**) of two real-world networks: (**a**,**b**) the resting-state fMRI network of human brain^36^ and (**c**,**d**) the global airline network from the website Openflight.org^37^. The assortativity coefficient is *r* = 0.3886 in panel a, and *r* = 0.0461 in panel c. The corresponding degree distributions are shown in the insets. Here, degree distribution in (**b**) is well fitted with Poisson law with $\xi =4.94$ (*p* > 0.99) and degree distribution in (**c**) is well fitted with power law with *γ* = 1.25 (*p* > 0.99). The graphs’ visualizations are obtained by Gephi software^41^ using the OpenOrd layout.

To give an idea of how our results are applicable to real systems, we compare our findings with the main features of two real-world networks, whose visualizations are given in Fig. 6a,c. In particular, we focus on two very distinct spatial connectivity cases: the functional resting-state fMRI macroscale network of the human brain (638 nodes, 18,625 links) published in ref.^36^, and the global airline transport network (2,939 nodes, 31,354 links) taken from the website Openflight.org, and largely analyzed in ref.^37^.

The first one is an example of a network whose structure is the result of several spatial restrictions, and is a weighted network distributed in 3D space. Figure 6b shows the correlation between each node degree and the average degree of its neighbours: it is clearly seen that the real structure is characterized by an assortativity mixing with *r* = 0.3886. The degree distribution (shown in the inset) is rather homogenous featuring a high average value $({d}_{a}\approx 43)$. These characteristics have a strong similarity to those reported in Fig. 4b,c. This fact is strengthening our presumptions about the spatial restrictions of the real network. When one focuses on clustering coefficient and efficiency (the formulas for weighted networks can be found in ref.^38^ and ref.^39^, respectively), one obtains rather high values: *C* = 0.4128 and *E* = 0.3224. This set of network characteristics is consistent with the border between two parameter regions of our model: at moderate values of $\beta \approx 0.3$ and small values of the density gradient, *λ*, the density of links is still high implying small shortest-path lengths and high clustering in the system.

As for the second case, the airline network is a clear example of a spatially-distributed network with a very large number of long-range connections. Such a graph is unweighted and undirected, allowing a more straightforward comparison with our model. The assortativity coefficient is very small (*r* = 0.461), due to the existence of hub-periphery connections. Looking at the corresponding (*d*_*i*_, *u*_*i*_) scatter-plot in Fig. 6d, one can observe a pattern which is very similar to the one reported in Fig. 4d,e. Furthermore, long-range connectivities determine here a power-law degree distribution (see inset), a fact that confirms the predictions made by our model.

Our results qualitatively agree also with a series of observation made in other spatially-extended networks. Indeed, we pointed out that a complex topology may emerge in association with local assortative behavior and long-distance connections to structural hubs. This pattern has been recently observed in spatial networks of cultured neurons^28^, in close relationship with the ability of neural networks to maintain an optimal and robust topology^11^. In particular, recent studies demonstrated that the formation of patterns of local cooperation between nodes, together with the emergence of a backbone of hubs in the network provide a structure much more stable and resilient (against external attacks) than simple assortative or disassortative mixing^40^.
